# Comparison of fixed and mobile-bearing total knee arthroplasty in terms of patellofemoral pain and function: a prospective, randomised, controlled trial

**DOI:** 10.1186/s12891-017-1635-9

**Published:** 2017-06-29

**Authors:** P. Z. Feczko, L. M. Jutten, M. J. van Steyn, P. Deckers, P. J. Emans, J. J. Arts

**Affiliations:** 1 Department of Orthopaedic Surgery, CAPHRI Research School, Maastricht University Medical Centre, P. Debyelaan 25, 6229 HX Maastricht, the Netherlands; 2 Reynaert Private Hospital, Maastricht, the Netherlands; 3 Department of Orthopaedic Surgery, Zuyderland Hospital, Heerlen, the Netherlands

**Keywords:** Total knee arthroplasty, Anterior knee pain, Mobile bearing, Fixed bearing

## Abstract

**Background:**

Despite growing evidence in the literature, there is still a lack of consensus regarding the use of the mobile-bearing (MB) design total knee arthroplasty (TKA).

**Methods:**

In a prospective, comparative, randomised, single centre trial, 106 patients with end-stage osteoarthritis of the knee were randomised to either an MB or fixed-bearing (FB) group to receive posterior stabilised (PS)-TKA using a standard medial parapatellar approach and patellar resurfacing with follow-up (FU) for 5 years. The primary outcome was anterior knee pain (AKP) during the chair rise test and the stair climb test 5 years after surgery. The secondary outcome was the ability to rise from a chair and to climb stairs, range of motion (ROM), Knee Society Score (KSS), RAND-36 scores and radiological analysis of the patellar tilt.

**Results:**

No statistically significant difference was found between the two groups at 5 years FU in terms of median AKP during the chair rise test and the stair climb test (*p* = 0.5 and *p* = 0.8, respectively). There was no significant difference in any of the other secondary outcome parameters between the groups at 5 years FU.

**Conclusion:**

A mobile-bearing TKA does not decrease AKP compared to fixed bearings.

**Trial registration number:**

ClinicalTrials.gov NCT02892838.

**Level of evidence:**

II

## Background

Total knee arthroplasty (TKA) is a successful surgical treatment for osteoarthritis of the knee [1–3]. This intervention results in excellent long-term survivorship [4–7] and marked improvement in functional capacity and quality of life for the patients [8]. However anterior knee pain (AKP) is present in 4 to 40% of all cases [9–11] independently of patellar resurfacing, restricting the patients in climbing stairs, rising from a chair, cycling, or, in worst case scenarios, walking normally. The causes of AKP are multi-factorial and can be divided into non-modifiable and modifiable factors [12, 13]. Non-modifiable factors are young age, female gender, ethnicity and low pain threshold [14–17]. Modifiable factors can be patient related, like anxiety, depression, pain processing problems [18, 19], muscle imbalance and dynamic valgus during gate [12]. A wide range of non-patient related, modifiable factors are published in the literature to explain and treat AKP after TKA [12–17]. Van Jonbergen [20] found inflammatory changes in the Hoffa and local peripatellar synovitis. Van Jonbergen and coworkers reported a positive effect on AKP by resection of the Hoffa and peripatellar synovectomy. Patellar clunk syndrome [21–23] and the degree of wear of the patellar cartilage [24] were also linked with AKP. There is also growing evidence that prosthetic design features such as the morphology of the anterior flange of the femoral component, gender femoral component, single or multi radius design, and post-cam mechanism can have an influence on AKP [14, 15, 25–29]. The literature mostly reports on surgery-related factors after TKA. The application of circumpatellar electrocautery does not lessen the incidence of AKP [30, 31]. Resurfacing the patella also remains controversial [32–34]. According to Heergaard [35] TKA leads in nearly all cases to different patellar tracking and increased patellofemoral contact pressures. In contrast to the healthy knee in which conformity between the articular surfaces is optimal, the patellofemoral contact zones are significantly reduced after TKA [35]. Restoration of the standard patellar thickness and central positioning of the patella may minimise the contact forces [36, 37]. There is good experimental and clinical evidence that poor femoral or tibial rotational alignment can adversely affect patellar tracking and kinematics [35, 38–40]. The question is how to achieve optimal tibio-femoral and patellofemoral kinematics.

The mobile-bearing (MB) design TKA was introduced in the United States in 1980 first with the meniscal bearing concept, followed by the rotating platform design. The MB-TKA was developed to reduce polyethylene contact stresses and wear resulting in a lower rate of aseptic loosening. The other design goal was to create a self-aligning nature for the implants to provide an improved, more natural prosthetic knee joint and alignment with better functional results [41–46]. The MB design TKA was theoretically a revolutionary and attractive concept, however the clinical benefit is still controversial. Most meta-analyses could not show any benefit for the use of the MB-TKA [42, 47–50] in terms of clinical scores, loosening, ROM, pain, complications, quality of life, patient satisfaction and revision rate. There is no data in the meta-analyses for MB- versus fixed-bearing (FB) TKA in terms of AKP. Theoretically the MB design offers the potential advantage of self-correction of a rotational mismatch between the tibia and femur providing an optimization of patellofemoral mechanics and a potential reduction in AKP [51, 52] Most studies examine the kinematics of the patellofemoral joint in MB-TKA. Stiehl et al. [53] suggested that the MB design may reduce the patellofemoral maltracking resulting from the femoral component malposition conditions. Colwell [54] stated that the MB design can compensate for the malrotation of the femoral component on a limited basis. Sawaguchi [55] found in an intraoperative study where the medial shift and lateral tilt of the patella were significantly smaller in MB-TKA compared with FB-TKA. Lower patellofemoral contact stresses were found in MB-TKA compared with FB-TKA, however both designs had increased contact stress compared with native knees [56]. The New-Zealand Joint Registry study found a higher rate of revision for secondary resurfacing of the patella in the FB-TKA group [57].

The aim of the study was to collect more clinical data for AKP in MB- vs. FB-TKA patients. A prospective, comparative, randomised, single centre, trial including 106 patients was performed to compare mobile-bearing (MB) and fixed-bearing (FB) posterior stabilised (PS) TKA with patella resurfacing at 5 years follow-up (FU).

The primary outcome was anterior knee pain during the chair rise test and the stair climb test 5 years after surgery. The secondary outcome was the ability to rise from a chair and to climb stairs, range of motion (ROM), Knee Society Score (KSS), RAND-36 scores and radiological analysis of the patellar tilt 5 years after surgery.

The null hypothesis was that patients in the MB-TKA group do not exhibit less AKP during rising from a chair or climbing stairs.

The alternative hypothesis (H1) was that patients in the MB-TKA group do exhibit less AKP during rising from a chair or climbing stairs.

## Method

### Trial design

A prospective, comparative, randomised, single centre trial that included 106 patients was performed to compare MB and FB PS-TKA with patella resurfacing at 5 years follow-up (FU). Patients with end-stage osteoarthritis of the knee were randomised to either an MB or FB group to receive PS-TKA using a standard medial parapatellar approach.

### Ethics, participant selection and consent

Ethical approval was obtained from the local ethical committee of Maastricht (METC 08–055), as part of the research program, “Should my knee rotate? A randomised controlled trial to compare fixed and mobile-bearing total knee arthroplasty using the Scorpio PS SuperFlex and Scorpio + PS Mobile Bearing knee systems”. Patients were randomised (random permuted blocks of changing size) in either the MB or the FB group. The randomization process was computer generated using SPSS software. The randomization scheme ensured that during the enrolment period the ratio of the number of cases in the two groups remained constant. A written informed consent was obtained from all participants. All data was collected at the Department of Orthopaedics of Maastricht University Medical Centre. All patients and the researcher, who collected the data, was blinded. The surgeons were not blinded (see also author’s contribution).

Trial Registration Number: ClinicalTrials.gov NCT02892838 Retrospectively registered (2 Sep 2016).

### Inclusion and exclusion criteria

Inclusion criteria included patients between 21 and 80 years of age who had an established diagnosis of knee osteoarthritis or post-traumatic arthritis requiring primary total knee replacement. Exclusion criteria included medio-lateral instability greater than 10 degrees, active inflammation or infection of the knee, and patients with diagnosed systemic disease (such as bone diseases, immunologically suppressed conditions, neuromuscular deficits, Complex Regional Pain Syndrome (CRPS) that would have affected the overall outcome of the study. In addition, patients were excluded if they we unable to receive a patella component (e.g., old patella fracture, too thin patella, etc.).

### Interventions (operative procedure)

The aim of the operation was to achieve neutral coronal limb alignment ±2° and a stable knee defined as having a maximum of 0–3 mm laxity of the collateral ligaments [58].

All knee surgeries were performed by two surgeons. A medial parapatellar approach was applied in all cases using a tourniquet. The rotational position of the femoral component was determined by using the *Whiteside’s line* and the transepicondylar line (TEA) [59, 60]. The rotational position of the tibial tray was determined by using the medial one third of the tibial tubercle [61, 62]. The tibial slope was corrected to 0 degrees. With both techniques, after determining proper prosthetic size, the collateral ligaments were balanced as required based on ligament tension assessed during functional testing of the prosthetic implant [63]. Patients younger than 70 years of age received cementless femoral and tibial components, while patients older than 70 years of age received cemented implants using Simplex-P (Stryker Howmedica Osteonics, Allendale, NJ USA) containing antibiotics. Cemented patellar surface implantation was performed in every case. In each case, a Scorpio (Stryker Howmedica Osteonics, Allendale, NJ USA) PS implant was used with fixed- or mobile-bearing inserts.

### Outcome measurements

Clinical outcomes were assessed by a blinded independent examiner. All clinical outcome parameters were assessed preoperatively and postoperatively at 6 weeks, 3 and 6 months, 1, 2 and 5 years.

The primary outcome was AKP during the chair raise test and the stair climb test measured on a visual analogue scale (VAS) [64, 65] 5 years after surgery. The secondary outcome was the ability to rise from a chair and to climb stairs, range of motion (ROM), Knee Society Score (KSS), RAND-36 scores and radiological analysis of the patellar tilt 5 years after surgery.

The chair rise test was assessed according to the Jones’ description [66]. The initial sitting position during the chair rise test was standardised. The patients were sitting on an adjustable chair with the hip and knee in 90° of flexion. The patients had to stand up from the chair without using their arms. The test was repeated five times and patients were asked to report pain and location of the pain. It was noted whether the patients were able to rise (yes or no) and the VAS was used to measure AKP.

In order to standardise the movement during stair climbing, the same stairs were used by each individual patient. The patients had to walk up and down 10 steps with alternating legs without using the handrail. It was noted whether the patients were able to rise (yes or no) and VAS was used to measure AKP. ROM was measured during physical examination using a goniometer according to the technique described by Norkin [67]. Intra-tester and inter-tester reliability was described by Brosseau [68], the reproducibility by Lenssen [69]. Knee Society Scores [70] and RAND-36 scores [71, 72] were also measured.

### Radiological evaluations

Standard plain radiographs with Merchant 30/60/90° views were performed preoperatively and postoperatively at 6 weeks, 3 and 6 months, 1, 2 and 5 years. The position of the patella was measured from the Merchant view producing an angle between a line through the most prominent parts of the femur and a line through the backside of the patellar component (cement–component interface) [73]. Mean and median values were used for further analyses.

### Statistics and sample size analysis

Descriptive statistics were used to summarise the data. Differences between ‘fixed’ and ‘mobile’ at 5 years were tested using Mann–Whitney U tests for continuous variables as normal distribution could not be assumed and chi-squared tests or Fisher’s Exact tests for categorical variables. Statistical analyses were performed using R version 3.3.1 (R Foundation, Vienna, Austria). *P*-values <0.05 were considered statistically significant.

A sample size estimation showed that 37 knees per group would be required to detect a clinically relevant difference of 1 point with a standard deviation of 1.5 points in the anterior knee pain VAS score, with an alpha of 0.05 and a power of 80%.

## Results

### Flowchart

**Fig. 1:**
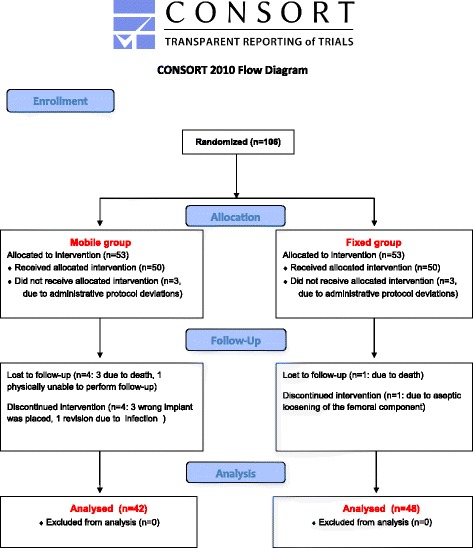
Flow-chart of participants. One hundred six participants were included for the study. Due to administrative protocol deviations, three patients from both groups were immediately excluded. Three additional patients of the MB group received wrong implant. Forty-seven patients in the MB and 50 patients in the FB group were available for the baseline data. Forty-two patients in the MB group and 48 patients in the FB group were available for the 5-year follow-up

One hundred six participants were included for the study. Due to administrative protocol deviations, three patients from both groups were immediately excluded. Three additional patients of the MB group received wrong implant. Forty-seven patients in the MB and 50 patients in the FB group were available for the baseline data. Forty-two patients in the MB group and 48 patients in the FB group were available for the 5-year follow-up (Fig. 1).

### Demographics

There was no significant difference between the two surgical groups with respect to gender, age, BMI, side of operation or primary and secondary outcome measurements (Table 1).

**Table 1:** Baseline characteristics and pre-operative values of outcomes

	Fixed (*n* = 50)	Mobile (*n* = 47)
BMI (kg/m^2^)	30.1 (±4.5)	28.7 (±4.2)
Side (L/R)	22/28	25/22
AKP during chair rise (VAS 0–10 median)	5 (0–10)	4 (0–8)
AKP during chair rise (yes/no %)	80/20	85.1/14.9
AKP during stair climb (VAS 0–10 median)	5.5 (0–10)	5 (0–9)
AKP during stair climb (yes/no %)	90/10	83/17
Ability to rise from a chair (able/unable %)	72/28	82.9/17.1
Ability to climb stairs (able/unable%)	97.9/2.1	95.3/4.7
Range of motion (ROM) (degrees)		
Flexion	110 (85–140)	110 (75–140)
Extension	−5 (−20–5)	−5 (−35–5)
Total	105 (70–140)	110 (65–140)
KSS		
Pain	48 (11–92)	49 (11–83)
Function	55 (0–80)	60 (0–90)
Total	101 (31–157)	106 (25–151)
RAND-36		
Physical functioning	30 (5–75)	35 (5–90)
Social role functioning	62 (0–100)	62 (0–100)
Physical role functioning	0 (0–100)	0 (0–100)
Emotional role functioning	33 (0–100)	50 (0–100)
Mental health	60 (4–100)	70 (8–96)
Vitality	50 (15–95)	55 (0–90)
Bodily pain	40 (0–80)	40 (0–80)
General health perceptions	60 (15–95)	62.5 (20–100)
General health change	50 (0–75)	50 (0–100)
Patellar tilt median (degrees)		
30 degrees flexion	2 (0–25)	2 (−1–13)
60 degrees flexion	2 (0–13)	2 (0–11)
90 degrees flexion	1 (0–11)	2 (0–10)
Patella tilt mean (degrees + SD)		
30 degrees of flexion	2.70 ± 4.06	3.0 ± 3.39
60 degrees of flexion	2.26 ± 2.72	2.72 ± 2.73
90 degrees of flexion	1.58 ± 2.17	2.29 ± 2.51

### Primary outcomes

At 5 years follow-up, median AKP scores during chair rise and during stair climb in the ‘fixed’ group were 0 (range 0–7) and 0 (range 0–8), respectively. In the ‘mobile’ group median pain scores during chair rise and stair climb were both zero (range 0–7). No statistically significant difference in anterior knee pain during chair rise (*p* = 0.5) and anterior knee pain during stair climb (*p* = 0.8) between the two surgical groups was found (Table 2). There was no significant difference between groups in terms of percentage of participants having AKP during chair rise (FB group 22% vs. MB group 14.9%, *p* = 0.3) or during stair climb (16% vs. 17%, respectively, *p* = 0.9).

**Table 2:** Primary and secondary outcomes

	Fixed (*n* = 48)	Mobile (*n* = 42)	*p*-value
Primary outcomes			
AKP during chair rise median (VAS 0–10)	0 (0–7)	0 (0–7)	0.5
AKP during chair rise (yes/no %)	22/78	14.9/85.1	0.3
AKP during stair climb median (VAS 0–10)	0 (0–8)	0 (0–7)	0.8
AKP during stair climb (yes/no %)	16/84	17/83	0.9
Secondary outcomes			
Ability to rise from chair (able/unable %)	97.9/2.1	95.2/4.8	0.6*
Ability to climb stairs (able/unable%)	97.9/2.1	97.9/4.8	0.6*
Range of motion (ROM) (degrees)			
Flexion	110 (80–130)	110 (85–130)	0.9
Extension	0 (−10–5)	−0 (−10–5)	0.7
Total	110 (70–130)	110 (85–130)	0.9
KSS			
Pain	94.0 (62–100)	95 (61–100)	0.8
Function	80 (30–100)	87.5 (5–100)	0.8
Total	174.5 (102–200)	178.5 (95–200)	0.8
RAND-36			
Physical functioning	55 (5–100)	55 (0–100)	0.6
Social role functioning	75 (25–100)	75 (0–100)	0.7
Physical role functioning	25 (0–100)	25 (0–100)	0.7
Emotional role functioning	100 (0–100)	67 (0–100)	0.3
Mental health	68 (4–100)	72 (20–100)	0.5
Vitality	60 (0–100)	65 (15–90)	0.9
Bodily pain	67 (12–100)	67 (0–100)	0.7
General health perceptions	65 (10–95)	55 (10–95)	0.6
General health change	50 (25–75)	50 (0–100)	0.6
Patellar tilt median (degrees)			
30 degrees flexion	1 (0–15)	0.5 (0–17)	0.4
60 degrees flexion	0 (0–15)	0 (0–20)	0.6
90 degrees flexion	0 (0–16)	0 (0–20)	0.4
Patellar tilt mean (degrees + SD)			
30 degrees flexion	2.56 ± 3.62	1.98 ± 3.58	
60 degrees flexion	1.96 ± 3.15	1.80 ± 3.82	
90 degrees flexion	1.81 ± 3.25	1.40 ± 3.42	

*Fisher’s Exact test

### Secondary outcomes

The ability to climb stairs and to rise from a chair, ROM, KSS scores, RAND-36 scores and patellar tilt were not statistically different between the two surgical groups (Table 2).

There was no statistically significant difference between the FB and MB groups in terms of ability to rise from a chair (*p* = 0.6, 97.9% vs. 95.2%, respectively) or ability to climb stairs (*p* = 0.6 97.9% vs. 97.9%, respectively).

There was no statistically significant difference between the FB and MB groups in terms of median ROM (*p* = 0.9, 110° (70–130) vs. 110° (85–130), respectively).

There was no statistically significant difference between the FB and MB groups in terms of total KSS and RAND-36 (Table 2).

There was no statistically significant difference between the FB and MB groups in terms of patellar tilt at 30°, 60°, 90° degrees of flexion (*p* = 0.4, 2.56 ± 3.62 vs. 1.98 ± 3.58, *p* = 0.6, 1.96 ± 3.15 vs. 1.80 ± 3.82, *p* = 0.4, 1.81 ± 3.25 vs. 1.40 ± 3.42, respectively).

## Discussion

Compared to the FB-TKA patients, patients in the MB-TKA group did not benefit from less anterior knee pain during rising from a chair or climbing stairs at 5 years follow-up in this study. This outcome is in line with the finding of previous meta-analyses, however AKP was not mentioned specifically. Only two meta-analyses [50, 74] reported lower pain scores in the MB group, but the quality of evidence was moderate to low [50, 74]. Price [75] and Breugem [76] reported lower pain scores in the MB group in the short term, but the same outcome was not confirmed in the long term [76, 77] nor did it differentiate AKP from general knee pain. The study by Biau [78] also showed a lower AKP in the MB group, however the difference was not statistically significant. This study showed 22% and 16% of patients had AKP during chair rise and stair climb in the FB group, meanwhile the AKP was 14.9% and 17% in the MB group during the same activities. Popovic [9] reported a much higher rate of AKP (49.2%) in posterior stabilised MB-TKA. The outcome was explained with the suboptimal trochlear design of the type of prosthesis. Wyatt et al. [57] reported a significantly higher rate of revision for secondary resurfacing of the patella in FB-PS-TKA designs compared with MB-TKA, which is not in line with the result of this study, however Wyatt reported a retrospective study.

AKP is known to cause the most problems in daily activities such as rising from a chair, or climbing stairs. Theoretically a larger percentage of patients who received MB-TKA would be able to rise from a chair and climb stairs compared with patients from the FB-TKA group, and while less patellar compression pain was expected in the MB group, it could not be confirmed. Little evidence can be found in the literature in terms of the ability to rise from a chair or climb stairs. Pais-Brito [79] found no differences between the MB- and FB-TKA groups in the ability to ascend and descend stairs. Woolson [80] stated that more patients in the MB group required aid to climb stairs compared with patients in the FB group, however this finding was statistically not significant. The meta-analysis by Smith [81] found no significant difference between groups based on nine studies.

Theoretically the MB design could lead to better ROM during daily activities [82]. We observed no difference in ROM between patients in either group. Most meta-analyses [42, 47, 48, 50, 81] also reported no significant differences between groups. Carothers [83] found no difference in ROM between groups, but the MB groups were significantly better in increase of ROM compared with the pre-operative function. Aglietti [84] found better ROM in the MB group while Haas [82] reported better ROM in the FB group. Kim [85] found minimally better ROM in the MB group although the difference was not statistically significant. The variation in design of the MB produced differences in ROM between the MB- and FB-TKA [49]. Since several MB designs are available (pure rotation, pure translation, combined rotation-translation and meniscal bearing) the results of meta-analyses can be influenced. A MB insert stops moving at flexion deeper than 90° and after this point the MB prostheses performs essentially as a fixed-bearing implant [49]. The question is how mobile is the bearing in MB prosthesis design during the stance phase of stair climbing and during rising from a chair if the flexion of the knee is less than 90°? The mobile-bearing insert can act as a fixed-bearing, but it is not proven. Studies utilising fluoroscopic techniques have demonstrated that knee joint kinematics are highly unpredictable in MB prostheses [86]. If mobile bearing insert act as a fixed bearing it could be an explanation why no differences were found between the two type of prostheses.

Stryker Scorpio PS MB and FB design was used in this study. The femoral components are the same in both prostheses with slightly different inserts. Both knees have single radius design and according to the manufacturer the Scorpio PS has great internal and external rotational freedom throughout the full range of motion. The design is not conforming between femoral component and tibial insert and as far as the authors know, there is no difference in conformity between the MB and FB design.

Most studies and meta-analyses [42, 47–50, 81, 83, 87, 88] reported no significant differences in clinical scores (KSS, HSS, WOMAC, OKS) between the MB and FB design TKA. Only two studies found significant differences in KSS is favour of the MB design TKA. The meta-analysis by van der Voort [49] reported significantly better physical SF-12 scores. The RAND-36 in our study was not different between groups.

No significant differences between the MB- and FB-TKAs were found in terms of mean and median patellar tilt in this study, which corresponds to Heinert’s results in a cadaveric study [89]. The rate of lateral releases was reported by Ferguson [90]. Lateral release was performed when tilting or subluxation was observed using the “no thumb” technique. The rate of releases was equal between the MB and FB groups. In contrast significantly smaller intra-operative lateral tilts of the patella were reported by Sawaguchi [55]. The average maximum contact stress of the patella was also significantly smaller. Skwara et al. [56] performed in vitro measurements of the patella. The MB design TKA showed evidently lower patellofemoral contact stresses than the FB design. Recent meta-analyses [42, 81, 88] reported no significant differences in lateral tilt of the patella between the MB and FB design TKA.

## Conclusion

No statistically significant difference was found between the FB and MB design PS-TKA in terms of patellofemoral pain and function at 5 years follow-up in this study.

### Limitation of the study

There are a few limitations to the study. There was no postoperative analysis on CT scan for the rotational position of the femoral component since it has a great influence on the patellar tilt. The authors also see a ceiling effect in the scoring lists and the question is whether they are sensitive enough to arrive at conclusions.

## Abbreviations

AKP: Anterior knee pain
CRPS: Complex regional pain syndrome
CT: Computer tomography
FB: Fixed-bearing
FU: Follow-up
KSS: Knee society score
MB: Mobile-bearing
PS: Posterior stabilized
RAND-36: Research and development survey
ROM: Range of motion
SPSS (software): Statistical package for the social sciences
TEA: Transepicondylar line
TKA: total knee arthroplasty
VAS: Visual analogue scale
WOMAC: Western Ontario and McMaster Universities Arthritis Index
